# Cytokine Profiles Contribute to Understanding the Pathogenic Difference Between Good Syndrome and Oral Lichen Planus: Two Case Reports and Literature Review

**DOI:** 10.1097/MD.0000000000000704

**Published:** 2015-04-10

**Authors:** Takashi Maehara, Masafumi Moriyama, Shintaro Kawano, Jun-Nosuke Hayashida, Sachiko Furukawa, Miho Ohta, Akihiko Tanaka, Masaki Yamauchi, Yukiko Ohyama, Tamotsu Kiyoshima, Seiji Nakamura

**Affiliations:** From the Section of Oral and Maxillofacial Oncology (TM, MM, SK, J-NH, SF, MO, AT, MY, SN); Section of Oral and Maxillofacial Surgery (YO); and Division of Maxillofacial Diagnostic and Surgical Sciences, Laboratory of Oral Pathology, Faculty of Dental Science, Kyushu University, Fukuoka, Japan (YK).

## Abstract

We described and analyzed the pathogenic difference between Good syndrome (GS) and oral lichen planus (OLP) in oral mucosa.

Good syndrome (GS) is a rare disease characterized by B and T cell immunodeficiency associated with hypogammaglobulinemia and thymoma. GS patients frequently develop oral lichenoid lesions with lymphocytic infiltration beneath the basal layer. Oral lichen planus (OLP) is a chronic inflammatory disease of the oral mucosa characterized by destruction of basal cells by Langerhans cells, macrophages, and T lymphocytes. Although the histological features of the lesions of both diseases are very similar, the pathogenesis of GS in the oral mucosa remains unknown. In this study, we thus investigated the expression of infiltrating lymphocyte subsets (CD3, CD20, CD4, and CD8) and T helper (Th) cytokines including interferon (IFN)-γ (Th1 type), interleukin (IL)-4 (Th2 type), IL-17 (Th17 type), and IL-10 (regulatory T cell type) by immunohistochemistry in buccal mucosa specimens from 2 GS patients compared with 15 OLP patients. All patients showed a predominance of CD3^+^ T cells over CD20^+^ B cells, and CD4^+^ Th cells over CD8^+^ cytotoxic T cells. This polarization was especially prominent in GS. IFN-γ and IL-10 were strongly detected in the infiltrating lymphocytes of all patients. However, IL-4 and IL-17 were detected in OLP patients only.

These results suggest that the pathogenesis of GS is different from that of OLP. GS is a unique inflammatory disorder characterized by dysfunction of Th2 and Th17 immune reactions via abnormal T–B cell interaction.

## INTRODUCTION

Oral lichen planus (OLP) is a chronic inflammatory disease that affects 0.1% to 4% of the general adult population.^1^ This disease is characterized by reticular white lesions with mucosal atrophy and erosions, usually distributed bilaterally on the buccal mucosa and occasionally in the tongue. Histopathologically, these lesions are characterized by a subepithelial band-like inflammatory infiltrate, variable numbers of intraepithelial mononuclear cells focused around basal keratinocytes, and destruction of basal cells. The pathogenesis has been determined immunohistochemically to involve Langerhans cells as well as infiltrating cells including both CD4^+^ and CD8^+^ cells. Infiltrating CD4^+^ T helper (Th) cells enhance expression of adhesion molecules and secrete cytokines depending on signals from the local microenvironment, which induce more Th cells to migrate, altering the balance toward pathology.^2^ Furthermore, we previously reported that Th cells are involved in the pathogenesis of OLP; in particular, we reported that the potential antigen recognized by these Th cells was derived from basal epithelial cells.^3^ Identification of the specificity of Th cells is one of the most important steps to reveal the pathogenesis and etiology of OLP.

Good syndrome (GS) is characterized as thymoma complicated with hypogammaglobulinemia and involves various immunodeficient conditions including depleted B cells, reduced T cells, and inversion of the CD4/CD8 ratio.^4^ It was first described by Dr. Robert Good in 1954.^5^ Although the pathogenesis of GS is still uncertain, a bone marrow defect impairing B-cell maturation has been suggested. Theodoros et al^6^ identified 89 GS patients presenting with autoimmune manifestations, such as pure red cell aplasia (34.8%), myasthenia gravis (15.7%), oral lichenoid lesions (12.4%), and other hematological abnormalities. Clinical examination of oral mucosa in GS patients showed painful erosion with a reticulated appearance on the lateral aspects of the tongue and the buccal mucosa,^7^ which is clinically similar to OLP. However, to our knowledge, no published reports have investigated the mechanism promoting oral mucositis in GS. Consequently, an understanding of the presence of infiltrating lymphocyte subsets in the lesions of buccal mucosa from OLP and GS patients is relevant to clarifying the difference of the onset and progression between these diseases.

## CASE REPORTS

### Case 1

**FIGURE 1:**
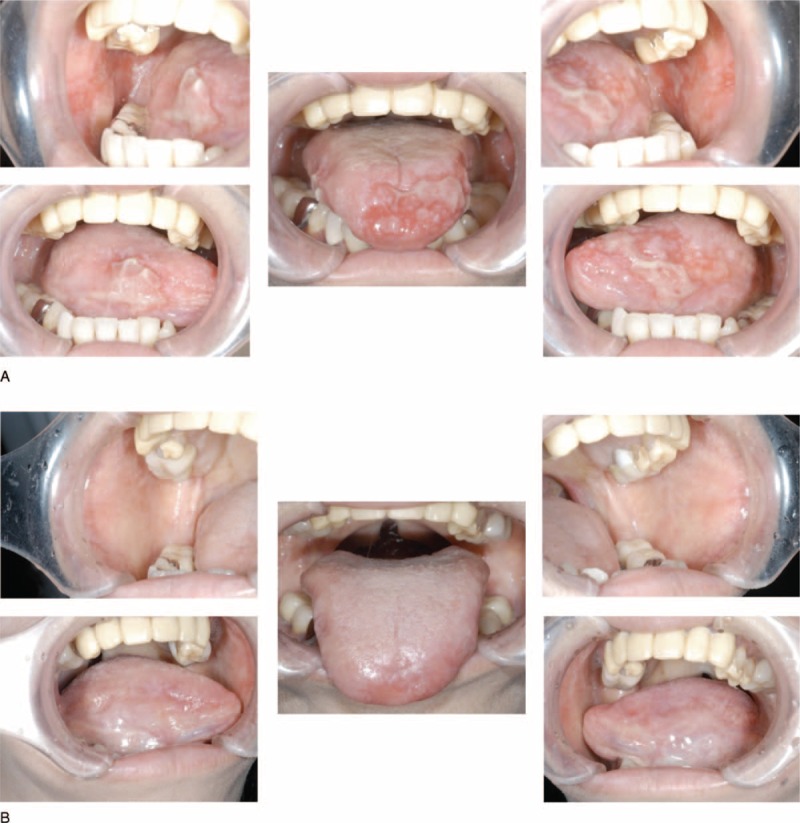
Clinical findings in oral cavity from patient with Good syndrome (GC) (Case 1). (A) Oral mucosa and tongue at the initial visit. (B) Oral mucosa and tongue after thymectomy.

**FIGURE 2:**
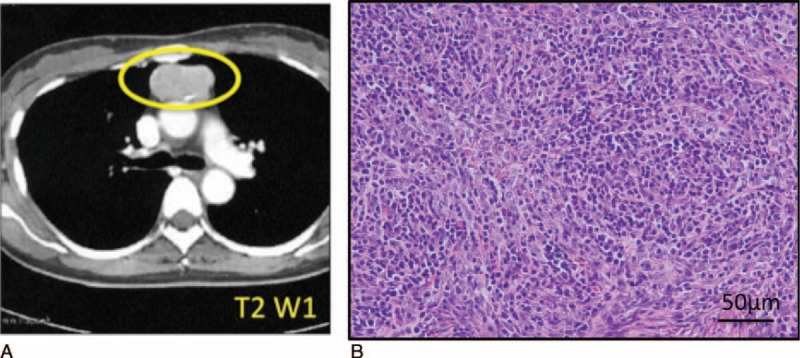
Imaging and histological findings in thymus gland from patient with Good syndrome (GS) (Case 1). (A) Computed tomography findings demonstrating mediastinal abnormality and mass in the anterior mediastinum (yellow circle). (B) Thymus gland specimen was stained with hematoxylin and eosin (HE). Scale bar, 50 μm.

A 48-year-old woman was referred to the Department of Oral and Maxillofacial Surgery, Kyushu University Hospital, in August 2008, for erosive and hyperkeratotic lesions of the oral mucosa and tongue recurring for over a year (Fig. 1A). She had no remarkable medical history except for hypoferric anemia. She had low hemoglobin concentration (8.9 g/dL), decreased hematocrit (29.1%), low erythrocyte indices (mean corpuscular volume, 71.0 fl; mean corpuscular hemoglobin, 21.7 pg), and white blood cell count of 4760 cells/mm3 (neutrophils, 70.3%; lymphocytes, 24.4%; eosinophils, 5.3%; basophils, 0.0%). All serum chemistry data were within normal limits. Her C-reactive protein concentration was 0.03 mg/dL. Serum antibody tests were negative for Dsg1, Dsg3, HIV, hepatitis B virus (HBV), and hepatitis C virus (HCV) antibody. Serum levels of IgG, IgA, IgM, and IgE were less than normal limits (516, 6, 14, and 20 mg/dL, respectively). Computed tomography (CT) and magnetic resonance imaging (MRI) findings raised suspicions of thymoma, and thymectomy and intravenous immunoglobulin were performed in January 2009. Pathological findings revealed World Health Organization (WHO) classification type AB thymoma (Fig. 2), which, combined with clinical features, resulted in a definitive diagnosis of GS. The erosive and hyperkeratotic lesions of the oral mucosa and tongue were significantly improved by the thymectomy and intravenous immunoglobulin (Fig. 1B).

### Case 2

A 52-year-old woman was referred to the Department of Oral and Maxillofacial Surgery, Kyushu University Hospital, in January 2014, for recurrent erosive and ulcerated lesions of the buccal mucosa and tongue recurring from 2 months ago. She had no remarkable medical history except for hypoferric anemia. She had low hemoglobin concentration (11.5 g/dL), normal hematocrit (35.7%), normal erythrocyte indices (mean corpuscular volume, 91.2 fl; mean corpuscular hemoglobin, 29.5 pg), and white blood cell count of 3280 cells/mm3 (neutrophils, 60.5%; lymphocytes, 21.0%; eosinophils, 9.6%; basophils, 1.0%). In serum chemistry, total protein and albumin were less than normal limits (5.5 and 3.6 g/dL, respectively), whereas the other data were within normal limits. Her C-reactive protein concentration was 1.11 mg/dL. Serum antibody tests were negative for Dsg1, Dsg3, HIV, HBV, and HCV antibody. Serum levels of IgG, IgA, and IgM were less than normal limits (466, 71, and 32 mg/dL, respectively). CT findings raised suspicions of thymoma, and thymectomy and intravenous immunoglobulin were performed in December 2014. Pathological findings revealed WHO classification type AB thymoma, and resulted in a definitive diagnosis of GS. The erosive and ulcerated lesions of the buccal mucosa and tongue were slightly improved by the thymectomy, intravenous immunoglobulin, and oral rinse of steroid solution.

As comparative subjects, 15 patients with OLP (3 men and 12 women; mean ± SD age, 58.1 ± 7.0 years) who were referred to the Department of Oral and Maxillofacial Surgery, Kyushu University Hospital between 2007 and 2011 were included in the study. Medical records were retrospectively reviewed after the diagnosis. There was no documented history of HIV, infection with HBV, HCV, sarcoidosis, or any other known immune-depressants for any patient.

Buccal mucosa specimens from 9 patients with OLP and the patient with GS were investigated histologically. Formalin-fixed and paraffin-embedded 4-μm sections of buccal mucosa were prepared and stained with hematoxylin and eosin (HE) for conventional histologic examination. The diagnosis of OLP was made by clinical features and histopathological findings. In all cases, the lesions showed the characteristic clinical features of the erosive form of OLP.

For immunohistochemical analysis, 4-μm formalin-fixed and paraffin-embedded sections were prepared and stained by a conventional avidin–biotin complex technique as previously described.^8–11^ The mouse monoclonal antibodies used to analyze lymphocyte subsets were anti-CD3, anti-CD20, anti-CD4 and anti-CD8 (Leu4, Leu12, Leu3a + 3b, and Leu2a, respectively; BD Bioscience, San Jose, CA). The polyclonal antibodies used to analyze cytokines were anti-IL-4 (clone: ab9622; Abcam, Cambridge, UK), anti-IL-10 (clone: ab34843; Abcam, Cambridge, UK), anti-IL-17 (clone: sc-7927; Santa Cruz Biotechnology, Santa Cruz, CA), and anti-IFN-γ (clone: ab9657; Abcam, Cambridge, UK). The sections were sequentially incubated with primary antibodies, biotinylated anti-mouse IgG secondary antibodies (Vector Laboratories, Burlingame, CA), avidin-biotin horseradish peroxidase complex (Vector Laboratories), and 3,3’-diaminobenzidine (Vector Laboratories). Mayer's hematoxylin was used for counterstaining. Photomicrographs were obtained using a light microscope equipped with a digital camera (CoolSNAP; Photometrics, Tucson, AZ). To quantify the IHC-positive cells, stained CD3, CD4, CD8, CD20, IFN-γ, IL-4, IL-10, and IL-17^+^ cells were counted in 1-mm^2^ sections from 5 different areas. Furthermore, the ratio of CD3^+^ to CD20^+^ cells and CD4^+^ to CD8^+^ cells was calculated. Evaluations of immunostaining were conducted by pathologists (TK and HS) who were blinded to information on the samples. Positive cells were counted randomly and the data of TK and HS were averaged.

The study design was approved by the Ethics Committee of Kyushu University, Japan, and all participants provided written informed consent (IRB number: 22–15), which was obtained from the patient for the publication of this report and any accompanying images.

### Histological Findings and Lymphocyte Subsets in the Buccal Mucosa Specimens

**FIGURE 3:**
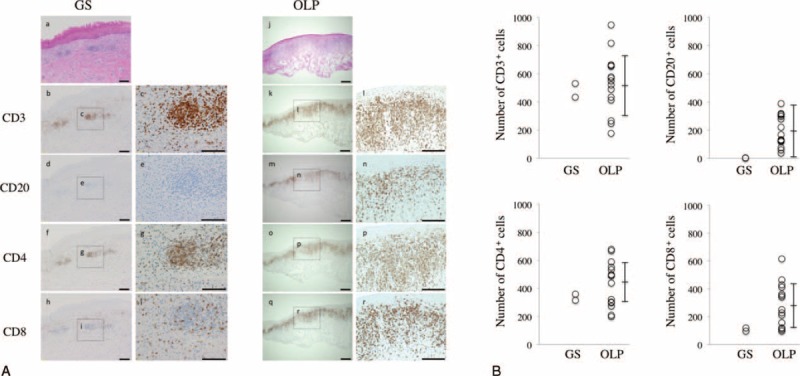
Lymphocyte subsets in the buccal mucosa from patients with GS and OLP. (A) Buccal mucosa specimens from representative patients with GS and OLP were stained with HE (a and j) and anti-CD3 (b, c, k, and l), anti-CD20 (d, e, m, and n), anti-CD4 (f, g, o, and p), and anti-CD8 (h, i, q, and r) monoclonal antibodies. Counterstaining with Mayer hematoxylin was subsequently performed. Scale bars, 100 μm. (B) Number of CD3, CD20, CD4, and CD8^+^ cells per HPF were counted in 1-mm^2^ sections from 5 different areas from patients with GS (n = 2) and OLP (n = 15). GS = Good syndrome, HPF = high-power field, OLP = oral lichen planus.

Representative histological findings in the buccal mucosa are shown in Fig. 3. Both GS and OLP patients showed typical band-like lymphocytic infiltration and diffuse infiltration into the connective tissue papillae and the lamina propria. The GS patient showed diffuse infiltration of CD3^+^ T cells in the lamina propria, whereas CD20^+^ B cells were rarely seen. In contrast, The OLP patients showed strong, band-like infiltrations of CD3^+^ T cells and diffuse infiltrations of CD20^+^ B cells in the sub-basal region. A slight predominance of CD4^+^ T cells over CD8^+^ T cells was observed in the OLP patients, whereas an increased predominance of CD4^+^ cells over CD8^+^ cells was seen in the GS patient (Fig. 3B).

### Distribution of Cytokines in the Buccal Mucosa

**FIGURE 4:**
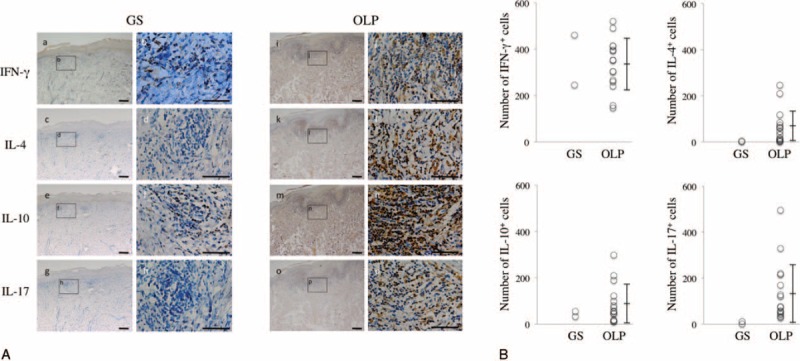
Distribution of T helper cytokines in the buccal mucosa from patients with GS and OLP. Immunostaining with anti-IFN-γ (a, b, i, and j), anti-IL-4 (c, d, k, and l), anti-IL-10 (e, f, m, and n), and anti-IL-17 (g, h, o, and p) monoclonal antibodies in buccal mucosa of representative patients with GS and OLP. Counterstaining with Mayer's hematoxylin (blue). Scale bars, 100 μm. (B) Number of IFN-γ, IL-4, IL-10, and IL17^+^ cells per HPF were counted in 1-mm^2^ sections from five different areas from patients with GS (n = 2) and OLP (n = 15). GS = Good syndrome, HPF = high-power field, IFN = interferon, IL = interleukin, OLP = oral lichen planus.

Given the results observed for lymphocyte subsets, it was of interest to investigate the involvement of CD4^+^ Th cells in the pathogenesis of these diseases. Th cell populations comprise functionally distinct subsets characterized by specific patterns of cytokine production. At least 4 subsets have been described including Th1, Th2, Th17, and regulatory T cells (Tregs), which produce IFN-γ, IL-4, IL-17, and IL-10, respectively. As shown in Figure 4, the specimens were immunohistochemically examined to evaluate the distributions of these cytokines in the buccal mucosa from GS and OLP patients. IFN-γ and IL-10 were strongly detected in the infiltrating lymphocytes of lamina propria from both GS and OLP patients. In contrast, IL-4 and IL-17 were also detected in the lymphocytic infiltration of lamina propria from the OLP patients, whereas they were rarely seen in the GS patient.

## DISCUSSION

GS presents with thymoma complicated with hypogammaglobulinemia, few or absent B cells, abnormal CD4/CD8 ratio, CD4 T cell lymphopenia, and impaired T cell mitogenic response as first described by Good et al.^5^ Almost all GS patients have reduced levels of immunoglobulins. B cells might exist in the spleen, lymph nodes, and other lymph tissues, ensuring immunoglobulin production, although at significantly reduced levels. Many cases of GS exhibit B- and T-cell lymphopenia, pre-B cell arrest, impaired maturation of erythroid and myeloid precursors, pure red cell aplasia, neutropenia, and eosinopenia. T cell abnormalities have also been observed in thymic tissue^12–14^ and it has also been suggested that GS might be caused by the breakdown of antitumor immunity from other tumors in addition to thymoma.^15^ Surgical removal of the thymoma is recommended. Although the most important indicator of long-term prognosis is completeness of tumor resection, the thymectomy does not often result in rescue of the hypogammaglobulinemia.^16^ Immunoglobulin replacement treatment including intravenous immunoglobulin has been reported to improve infection control, reduce hospitalization, and decrease the use of antibiotics.^17^ Although a temporary improvement is obtained, GS continues to result in a poor prognosis because of the risk of opportunistic infection.

We have previously reported that Th cells are involved in the pathogenesis of the disease process in OLP and the potential antigen recognized by these Th cells was derived from the basal epithelial cells in OLP lesions.^3^ In this study, CD3^+^ and CD4^+^ T cells were mainly distributed throughout both the connective tissue papillae and the lamina propria from both GS and OLP patients. Th1 cells have previously been shown to be involved in the pathogenesis of the disease process in OLP.^3,17^ We detected expression of the Th1-type cytokine (IFN-γ) in the lymphocytic infiltration of the lamina propria from GS and OLP patients, consistent with these reports. As Th2 cells have been implicated in the induction of B cell abnormalities, Th2 cells might also have a deleterious effect in OLP. Furthermore, Foxp3^+^ Tregs in both the lesions and peripheral blood of tissues are known to be involved in the pathogenesis of OLP.^18^ Tregs are essential for the maintenance of immunological self-tolerance and immune homeostasis. In this study, although expression of the Treg-type cytokine (IL-10) was detected in the lymphocytic infiltration of lamina propria from GS and OLP patients, expression of the Th2-type cytokine (IL-4) and the Th17-type cytokine (IL-17) was detected in the OLP patients, but not the GS patient. Furthermore, our results showed that CD20^+^ B cells were not detected in the buccal mucosa of the GS patient. Possible explanations for these results include: disturbance in B cell lineage differentiation because of putative bone marrow-derived humoral factors; and T cell dysfunction causing disturbed B-cell lineage differentiation.^19^ According to the latter hypothesis, T cell dysfunction could result in both thymoma and hypogammaglobulinemia, and may explain the maintenance of GS symptoms after thymectomy.^20^ In contrast, oral lichenoid lesions of the GS patient improve after thymectomy.

We are aware of no published reports that describe the selective localization of Th subsets in lesions from GS patients. Evaluating greater numbers of patients with GS will help to elucidate the clinical and histological differences between OLP and GS, which may lead to clarification of the pathogenesis of GS.
